# Putting Knowledge into Practice: Low-Income Women Talk about Food Choice Decisions

**DOI:** 10.3390/ijerph17145092

**Published:** 2020-07-15

**Authors:** Shelly M. Palmer, Simon T. Knoblauch, Donna M. Winham, Molly B. Hiller, Mack C. Shelley

**Affiliations:** 1 Food Science & Human Nutrition, Iowa State University, Ames, IA 50010, USA; s-palmer94@live.com (S.M.P.); simonk@iastate.edu (S.T.K.); molhiller@gmail.com (M.B.H.); 2 Political Science and Statistics, Iowa State University, Ames, IA 50010, USA; mshelley@iastate.edu

**Keywords:** food security, African Americans, diet, poverty, qualitative research, nutrition assistance, attitudes

## Abstract

Insights into barriers and facilitators for healthy eating are needed to improve low-income women’s diets and to decrease disease risk. The study objectives were to explore women’s qualitative perceptions of influences on their food choices such as food security, their knowledge of nutrition-related health risk factors and self-efficacy for diet change, and their dietary intakes in practice. Thirty-six women, aged 19–50, who were eligible to receive income-based assistance were recruited in central Iowa. Focus group discussions on defining healthy foods, influences on food choice, and nutrition information sources were analyzed using a socioecological model framework. Demographics, nutrient intake estimates, food security status, health behaviors, and self-efficacy for nutrition behavior change were collected by survey. Most participants were White (61%), single (69%), food insecure (69%), and living with children (67%). Few women met dietary recommendations. Barriers to healthy eating include cost, convenience/preparation time, family taste preferences, and limitations of federal food assistance programs. Facilitators are high self-efficacy for nutrition change and health knowledge on average. These results challenge the strategy of using nutrition education to improve healthy eating and instead show that intervention messaging should focus on limited, achievable steps to improve dietary choices that fit within cost, convenience, and taste constraints.

## 1. Introduction

Despite public health promotions to improve nutrition and increase physical activity, much of the United States (US) adult population has difficulty meeting health goals [1]. Few Americans meet daily fruit (12.2%) or vegetable (9.3%) intake recommendations [2]. Eighteen percent of Americans rate their health as “fair or poor”, while 10% have type 2 diabetes, and 42.4% are considered obese [1,3]. Dietary patterns were associated with almost half of all cardiometabolic deaths in a recent US national survey [4]. While most Americans have room for improvement, those with low socioeconomic status have increased health risk factors and poorer disease outcome [5].

Low-income populations suffer from multiple economically related barriers to healthy eating. Beyond the lack of finances, eating a well-balanced diet to curtail long-term chronic disease risks is only one of many complex priorities [6]. While nutrition knowledge and education are important, food choice decisions are heavily influenced by socioecological factors that may or may not be within an individual’s control [6,7,8,9,10]. Reducing grocery expenditures by eating less, buying cheaper products, self-provisioning, or seeking alternative sources of free food are common methods for coping with economic strain and can eclipse preferences for nutritional health [9]. Foods that satisfy taste and cultural preference may be more important than enhancing diet quality [10]. Prepackaged or convenience foods are increasingly popular due to time constraints and/or lack of cooking skills. They are often less expensive than fresh ingredients and are designed to have high taste appeal [9]. Thus, barriers to healthful food consumption may include cost, family preferences, and taste [6]. Other potential influences are an individual’s resistance to change or preference for the familiar [10].

Food insecurity is defined as “reduced quality, variety, or desirability of diet” and can include incidents of reduced intake [11]. In 2018, the prevalence of food insecurity was approximately 9% in Iowa and 11% in the US overall [12,13]. However, these state-level and national-level statistics can miss the subtlety of regional and ethnic differences at a smaller neighborhood scale [14]. Food-insecure individuals may have altered dietary patterns such as skipping meals and eating less than desired or needed for health [11]. Less healthy dietary patterns such as higher intake of refined grains, added sugars and fats, and lower fruit and vegetable consumption may occur [15]. These poor dietary conditions, in addition to environmental and socioeconomic circumstances, can lead to higher risk for chronic diseases, and subsequent increased health care costs [16].

Not just acute hunger but food insecurity can have long-lasting implications for different members of a household. Early childhood is a critical period of development, and future dietary practices are established in infancy [17]. Experiencing food insecurity in childhood has been linked to overweight and compensatory feeding habits in families. While social roles have become more diverse, women are frequently responsible for most of the food and meal decisions in low-income households [18]. Thus, they are an influential force in shaping food preference development for children, and food choices for their partners, family, and friends [17,18]. Furthermore, women experience higher rates of food insecurity compared to men in both non-married and married households [19].

Like other Midwestern states, fruit and vegetable consumption in Iowa is lower than nationally, with 11.7% and 7.0% meeting recommendations, respectively [20]. Approximately 15% of all Iowans self-reported their general health to be “fair or poor” [3]. As of 2019, Iowa’s adult obesity prevalence was 35% and the type 2 diabetes prevalence was 10% [12,21]. However, for households earning less than $15,000, 40% of respondents reported their health as “fair or poor,” 50% of adults were obese, and 16% had type 2 diabetes [12,21].

Using a mixed-methods approach, the research objectives were to explore low-income women’s perceived influences on their food choices, such as food security, their knowledge of nutrition-related health risk factors and self-efficacy for diet change, and their dietary intakes in practice. This novel approach may improve understanding for behaviors in order to create tailored behavioral interventions beyond traditional nutrition education alone. A subset of this study specifically examining barriers to pulse (beans, peas, lentils, and chickpeas) consumption was reported elsewhere [22]. Data were analyzed to evaluate the congruence between such knowledge, perceptions, and practices, and an improved understanding of this interplay can inform the development of effective nutrition education and messaging that account for real-world constraints.

## 2. Materials and Methods

### 2.1. Survey Instruments and Focus Group Moderator Guide

Prior to beginning the focus group discussion, participants completed a multi-part survey composed of published validated questions. Most demographic questions (age, race/ethnicity, marital status, employment, education, and household composition) were from the Expanded Food and Nutrition Education (EFNEP) program [23], with marital status and education level drawn from the American Heart Association Women’s Survey [24]. The Behavioral Risk Factor Surveillance Survey (BRFSS) was used for employment and health risk factor questions (BMI, physical activity, smoking, alcohol use) [25]. Self-efficacy toward nutrition behavior change was assessed by four questions [26]. The 6-item USDA Core Food Security Module (CFSM) [27] estimated food security. The Block food frequency screeners which consist of 17 foods for fat, and 10 items for fruits, vegetables, and fiber, were used for dietary intake proxies [28]. Scores were generated by entering participant consumption frequencies into the online form [29].

The semi-structured focus group moderator guide incorporated literature review, previous research [30,31], and exploratory concepts. The socioecological model (SEM) framework guided structure of the variables for inquiry [32,33]. The SEM considers the effects of overlapping spheres of influence within an individual’s environment, including intrapersonal determinants, interpersonal social networks, and policy such as nutrition assistance programs and their nutrition messaging. The main themes of inquiry at the intrapersonal level of the SEM included women’s views of healthy foods, nutrition knowledge, and interactions of nutrition and disease. Interpersonal influences on food decisions focused on family and friends. Policy factors included sources of nutrition information and education, utilization of food assistance programs, interactions with health professionals regarding diet, and awareness of public health campaigns, e.g., MyPlate. The research team moderator (S.M.P.) and main field observer (M.B.H.) had previous qualitative methods research experience and coursework. Four nutrition education researchers reviewed the focus group guide for content and construct validity for fit with the SEM. Seven Cooperative Extension Expanded Food and Nutrition Education Program (EFNEP) educators participated in the pilot test of the survey and interview guide. Their suggested modifications improved flow and clarity. The full survey and focus group moderator guide are available upon request or from the published thesis [34].

### 2.2. Study Participants, Recruitment, and Procedures

Low-income women were recruited through flyers at community agencies and Cooperative Extension programs in Ames and Des Moines municipalities, e.g., YMCA, public libraries, Special Supplemental Nutrition Program for Women, Infants, and Children (WIC) offices, health clinics, and food pantries. Interested parties were screened for eligibility during a telephone interview (criteria: female, aged 18–50 years, English fluency, receives income-based assistance). After clustering by preferred geographic locations, times and dates available, focus groups of 5–10 women were contacted to schedule attendance. The same researcher (S.M.P.) moderated all focus groups, while two other team members recorded field notes. Sample size determination was based on focus group data themes reaching saturation, or the point where no new emergent views were found. Most sessions lasted approximately 75 min and ranged from 45 to 120 min. Participants received $40 cash for completion of the study. The Iowa State University Institutional Review Board (#17-292) approved study procedures.

### 2.3. Data Transformations and Analysis

Survey data were entered and analyzed in SPSS v. 24 (IBM, Armonk, NY, USA). Data were examined for normality. Food security raw scores were categorized as high, low, and very low food security per the CFSM instructions [27]. Variables were compared by dichotomous categories of food security (secure/insecure). Comparisons by bivariate marital status (single/partner), presence/absence of children in the household, and race (White vs. African American) were made as a check for possible group differences or confounders. Although the sample size was relatively small for quantitative modeling, a logistic regression analysis was performed to predict food security status.

Digital audio recordings of focus groups were transcribed verbatim internally. Transcripts were independently coded by two trained qualitative researchers (S.M.P., M.B.H.) using NVivo software v.11.0, (QSR International, Burlington, MA, USA). Initial coding followed the focus group guide questions with added codes from themes voiced by the participants using a grounded theory approach [35]. Inter-rater reliability (99.59; kappa 0.83) was high after reaching consensus for final code allocation.

## 3. Results

### 3.1. Quantitative Survey

Fifty-four women inquired about the project during recruitment. Of these, eight were not eligible, four declined to schedule, and six did not attend a scheduled focus group. Sixty-one percent were White and 39% were African American. One White woman self-identified as Hispanic. Most women were had more than a high school education (58%), were single (69%), employed (50%), and food insecure (69%). Thirty-seven percent had an annual income of less than $10,000 (data not shown). Sixty-seven percent of women had children under age 18 in the household. Seventy-two percent of the women used one or more nutrition assistance program. The Supplemental Nutrition Assistance Program (SNAP) was the most utilized (47%) followed by WIC (36%) and school nutrition programs (31%) (Table 1). Program usage was significantly greater (*p* < 0.001) in food-insecure households, and those with children.

A logistic regression model was completed to ascertain whether eight variables (self-efficacy score, income, race, marital status, education, employment, smoking, and presence of children in household) could be used to predict food security status. The model correctly predicts 88.6% of reported food security status, including 22 of 24 (91.7%) instances of food insecurity and 9 of 11 (81.8%) instances of food security. Self-efficacy total score (*p* = 0.025) and employment status (*p* = 0.043) are statistically significant positive predictors. Logistic regression fit metrics indicate that the predictive model performs well (Cox and Snell pseudo-*R*^2^ = 0.455 and Nagelkerke pseudo-*R*^2^ = 0.639. The model with odds ratios for each variable is in the Appendix A Table A1.

Thirty-nine percent of the women stated that their health was fair to poor (Table 2). Approximately 77% were classified as overweight or obese by their BMI [36]. On average, women participated in moderate-intensity physical activity 3.5 days per week [37]. Food-secure women exercised more days than food-insecure women (4.6 vs. 3.0; *p* = 0.014). The majority of women did not smoke (72%). Forty-four percent reported drinking alcohol less than two times per year, but 19% drank at least once per week. Based on the dietary screeners, 64% of the women had a high (35–40%) or very high (40–50%) percentage of their total daily calories coming from fat. Sixty-nine percent consumed less than five servings of fruits and vegetables per day, and 83% did not attain 20 g of dietary fiber per day [28,29].

Summaries of self-efficacy Likert-type responses (very uncertain, rather uncertain, rather certain, and very certain) to five statements on managing a healthful diet are presented in Table 3 [26]. Participants with children were less likely to be certain they could make a detailed plan (*p* = 0.037). Food-insecure women were less certain that they could develop routines to stick to healthy foods (*p* = 0.044). While not significantly different, more women were uncertain they could make changes without the support of others. The self-efficacy score could range from 0 to 20, and the mean was high for the sampled women at 15.1.

The individual food items that comprised the dietary screeners are shown in Table 4 [28,29]. There were no differences for food items by food security, marital status, or children in the household. African American women reported eating fried chicken (*p* = 0.001) and bacon/sausage (*p* = 0.021) and drinking fruit juice (*p* = 0.033) significantly more often than White women.

### 3.2. Qualitative Results

#### 3.2.1. Definition of Healthy Foods

In describing their intrapersonal views and definitions of healthful foods, most women showed awareness of common nutrition advice and current dietary recommendations (e.g., lower fat and salt, increased intake of whole grains, everything in moderation). Some described healthy foods in terms of fresh fruits and vegetables and the way a well-balanced diet made them feel. Others discussed healthy eating in general terms.

*“Healthy includes a lot of things, but healthy foods are a balance like vegetables, fruits, and dairy … it makes you feel totally better, you have more energy and you don’t feel as old.”*

[age 31]

*“To me healthy is like a lifestyle or like a mental health thing—like it’s a balance between your choices and how you feel and your happiness …”*

[age 35]

Participants demonstrated some awareness of popular food trends to promote health. These included a plant-based diet, products like kombucha tea and probiotics for digestive health, and infusing water with fruit to make it more appealing for consumption and as an alternative to other beverages like sugar-sweetened sodas.

#### 3.2.2. Family Member Influences on Food Choices

The influences of household members on food choices was clearly voiced in all focus groups, especially regarding children’s influences on the purchasing habits of the mother and the food type provided during mealtimes.

*“… If I’m feeding myself, I’ll get something to shove in the microwave, I don’t care. But if I’m feeding my kids I’ll buy something else that they can eat, or would eat.”*

[age 31]

*“I don’t buy the foods I want … they ask me and I’ll take them to the store because they are teenagers, 17 and 14, I don’t know what they will want to eat. That’s why I waste my food stamps … because they want different stuff and it costs different prices and they don’t understand we are working on a budget.”*

[age 47]

Women frequently stated that their children or adult partners influenced meals. Changing foods to suit preferences of the child or making an alternative meal to accommodate the child’s taste was frequently voiced. Alternatively, attempts to control the healthy eating of others in the household by eating less meat were met with resistance.

*“I pretty much go off what the kids want to eat. When I cook a meal, they don’t want to eat it. They don’t.”*

[age 47]

*“I don’t buy a lot of processed foods because I know they are full of all those things, I try to cook without oil. I cut out butter … My husband’s always complaining but I keep the salt to absolute minimum and he’s always adding more to it.”*

[age 35]

*“*[If cooked vegetarian meals] … *My husband would ask, where’s the meat?”*

[age 31]

*“I just make sure that I buy bulk meat. That way … if we do run out [of food] we still have the main course. Honestly, it’s not really healthy to eat all of this meat. … You can’t keep the kids full without it being the main dish.”*

[age 29]

Some women stated that having children had positive influences on their own food behavior. The women mentioned changing regular aspects of their diets in order to provide a positive influence for their children.

*“Before I had kids I used to be a big food junkie, lots of candy, lots of soda, but then I realized that in order to raise healthy kids I would have to start changing my lifestyle.”*

[age 35]

*“When we were in college, I would say that we would not eat as well, like pizza, hot dogs and cheaply. Obviously now that we have kids we try and have more balanced meals and regular meals, not just sporadic like stop in and get a slice of pizza.”*

[age 31]

One woman mentioned wanting to eat healthy foods so her breastfed daughter would have optimal access to nutrients.

*“Because right now I have a baby of seven months old and I am breastfeeding her so it’s very important to me to have a very good and nutritious food. Because if I am taking the good one then she will be taking the good one through me.”*

[age 32]

#### 3.2.3. Barriers to Healthy Eating

Societal constraints on healthy eating at the intrapersonal level often related to the need for foods that were quick and convenient to prepare. Lacking sufficient time to prepare home-cooked meals that also appealed to family members was given as a common barrier among some participants. However, it is valuable to note some of the women shared positive experiences with convenient time-saving tools for meal preparation (e.g., crockpot, slow cooker, and Magic Bullet blender).

*“My problem is once I’m hungry, I want something fast. Like I don’t want to have to cook, you know the quickest possible thing that sounds good.”*

[age 28]

*“It’s hurry and cook dinner and get them all ready for bed … and so a lot of times it’s not always about the healthiest, it’s about what they are going to eat because sometimes you can fix something that’s healthier and they are going to look at it and be like, “I don’t like it.” … It’s about what they are going to eat and what I can get done in a decent amount of time.”*

[age 31]

At the community or policy level, several participants perceived that healthy foods available for purchase in retail stores were not affordable, or foods available from food assistance programs were not as appetizing.

*“The stuff that’s good for you is too expensive. You can’t afford [it] so you have to go back to what you can afford. That’s what you do, especially when you have children.”*

[age 49]

*“I would love to be able to afford a healthy meal and know what is healthy. I have an idea of what is healthy but to actually fix it and make it taste good … there’s a lot of things out there that are healthy. It’s expensive and it doesn’t taste good to me.”*

[age 39]

Focus group participants spoke about food security and coping strategies at several points during the discussion as barriers to healthy eating. With tight financial resources, women felt pressure to purchase enough food to last the entire month and often had to compromise quantity over nutritional quality. Some coping strategies cited included skipping or downsizing meals, frequenting food pantries, or utilizing funds appropriated for other bills to pay for groceries. Canned and frozen packaged foods were purchased since they have a longer shelf life and are convenient. Signs of food insecurity recurred throughout the discussions as one woman mentioned feeding her children took priority over feeding herself.

*“It got to the point where I had to stop eating so we would have enough. So now half the time I don’t feel hunger because I got so used to not eating as much.”*

[age 35]

#### 3.2.4. Utilization of Federal Nutrition Programs

In each of the seven focus groups, at least one woman mentioned receiving federal food assistance. Several women voiced frustration regarding these funds not lasting the entire month, wanting to purchase healthier foods but not being able to afford them, and restricted food offerings under SNAP and WIC policy guidelines.

*“Tomorrow is payday … we end up eating frozen pizza for supper, or pancakes, because the fresh fruits are long gone and [we are] limited on what’s left in the freezer.”*

[age 48]

*“And they looking at me because there is nothing in the refrigerator, that’s because you ate it all at the beginning of the month.”*

[age 47]

*“It’s more expensive to buy the healthier things so the food stamps they don’t go that far anyway but they can go further if you are buying things that are not as healthy.”*

[age 21]

*“My daughter likes Cuties, the little mandarin oranges, they’re like $6 a bag. I can’t afford nothing like that, even though it’s good and healthy … You know when you only get so many food stamps a month and you’re disabled … I mean we want to eat healthy, both of us are overweight, but it’s so expensive. Bananas, I get a lot of bananas because they are pretty cheap.”*

[age 39]

#### 3.2.5. Nutrition Information and Health Professionals

The internet was the most frequent source of nutrition information for most women. Participants reported using the internet for simple recipes, as well as applications like Pinterest for meal ideas. A majority of participants indicated they were familiar with the MyPlate diagram to guide food choices through previous Extension and WIC programming. However, when asked to discuss the five food groups, some struggled to think of specific food items from each group. Several participants deemed the WIC nutrition education vital to the health of their child and communicated trust in the credibility of the program’s educators.

Participants mentioned conversations with registered dietitians and doctors to improve their health status. Several stated they did not follow recommendations given because the goals seemed unrealistic. They perceived that nutrition professionals suggested too many restrictions and/or behavior changes. These overwhelming recommendations resulted in noncompliance in overall diet behavior.

*“I went to a dietitian … and I didn’t like what they were telling me so I never went back. You’re not supposed to eat this much and you’re not supposed to do that … I’m so used to how I make my food and I don’t know how to change and [still] like it.”*

[age 39]

*“She’s like, “Well eat more vegetables.” So I eat corn and green beans, and my doctor’s like, “The two most unhealthy vegetables are the ones you like.” And I’m like, “You told me to eat vegetables and that’s what I’m doing.”*

[age 31]

#### 3.2.6. Links between Nutrition, Health, and Chronic Disease

A few participants mentioned feeling in a “rut” with their current life and not having the resources or energy to pursue healthier diets and more physical activity. They recognized nutrition played an important role in maintaining and supporting overall health but stated it was often less a priority than imperative daily tasks. Not having the drive or consistency required to eat a nutritious diet on a regular basis was a recurring theme across the focus groups.

*“It’s just a frustrating world and there’s a lot that goes into living … I have a lot of issues in my life that I need to deal with [and] it’s just one step at a time and it’s like who is going to help me make those changes? Because I can’t do everything myself, I get overwhelmed, you know going to the doctor, being a single mom, pay[ing] rent … it’s something that I don’t feel like would fit into my life right now.”*

[age 39]

*“You feel terrible when you are not eating and drinking right. I used to drink water and I felt so good. Now I know what I am supposed to do but I have no energy or care to do it.”*

[age 35]

*“We just do a lot of tacos and honestly a lot of processed foods. Like pizza rolls because it’s easy, they like it … It’s just easier. Even though we’ve gained a lot of weight from it, it’s just easier … I feel guilty every single time but it’s more convenient and my kids like it … And it takes less time to prepare with my life … I am going through school also with everything, once things die down, I will be able to be healthier and plan more but for now I don’t plan as much.”*

[age 35]

Several women voiced knowledge of how food intake influenced their weight and health. There was recognition that lifestyle, including diet, both contributed to disease risk and could alleviate chronic conditions. While some participants indicated they had taken steps to improve their health through diet modifications, more often than not they felt barriers made compliance difficult.

*“I’m already kind of border line high blood pressure. So you know I just took out salt.”*

[age 29]

*“I try not to eat so much meat, because there is a lot of research in regards to colon cancers and you know that’s scary to me … I think about things that are substitutes like quinoa and other different things like that, sorghum, but it’s not easy to cook with them.”*

[age 34]

*“I can’t eat white potatoes, because I am diabetic but I still eat what I’m not supposed to because it’s cheaper.”*

[age 39]

Inquiries regarding health and dietary changes led to most focus groups discussing chronic conditions, primarily type 2 diabetes, high blood pressure, elevated cholesterol, obesity, and iron deficiency anemia. Other conditions mentioned were neuropathy, uric acid buildup, ulcerative colitis, cancer, and orthopedic issues. Although several participants with chronic conditions displayed knowledge of the benefits of healthy eating, they stated various barriers to managing their health through lifestyle means.

*“I’m type 1 diabetic, I have been since 2003, and I’m pretty much non-compliant, probably more detrimental to my health. I take 6–9 insulin shots per day to my gut … but what I eat should be stricter than what I do.”*

[age 39]

*“My daughter and I do admit we need to eat healthier, and me being [type 2] diabetic … for a healthy meal at a store … would probably cost three times more than getting ramen noodles, a microwave meal, or a frozen pizza.”*

[age 39]

*“With our health issues, my mom’s like, “If it’s good you can’t have it … you’re looking at your future, you’re going to have a stroke in like 4 years if you don’t get yourself together. You need to stop eating sugars, and sodas, everything, the starches.” Everything I’ll eat is not good for me.”*

[age 31]

## 4. Discussion

This research examined how low-income Iowa women perceived influences on their food choices alongside their definitions of healthy foods, and food intake, food security level, health risk factors and self-efficacy for diet change. Quantitative and qualitative results from the study highlight discordance between health knowledge and health behaviors. These findings reinforce the need to investigate situation-specific perspectives before launching public health interventions [8,30,33].

Self-reported dietary intake reflected less than desirable consumption patterns and intakes inconsistent with dietary recommendations. According to the dietary assessment screeners, fat consumption was high, and only 30% of the participants consumed five servings of fruits and vegetables per day. Yet the women indicated high self-efficacy for maintaining a healthy diet. In contrast, a survey in neighboring Iowa communities of low-income Hispanic and White women found they were more uncertain about their abilities to sustain behaviors [31]. This contrast suggests the focus group participants were confident in their ability to eat healthfully under ideal circumstances, but in reality, are stymied by common barriers such as time, affordability, household member influences, or other priorities. In fact, women with children and/or food insecurity were less certain than their peers about making detailed plans or keeping to routines. Anxiety over the likelihood of worsening chronic conditions did not appear to be a strong motivator in pursuing a healthier diet either. Participants did not necessarily revise priorities if faced with chronic disease conditions affected by lifestyle choices. It is possible that perceived conflict between knowledge and social relationships is resolved in favor of the latter and that actions are mediated by this cognitive dissonance [38].

While Iowa’s food insecurity prevalence is approximately 9% [3], the focus group participants had a much higher rate at almost 70%. The logistic regression model and the qualitative responses suggest that higher self-efficacy, as well as being employed, enable women to be more food secure. These factors are synergistic, and for many women getting ahead is difficult because of limited resources. Concern about healthier eating habits may not be an actionable goal [39].

Women discussed how having children brought about healthier diet changes for them. In cases of pregnancy and breastfeeding, women were interested in eating better so their infants would benefit. Similar findings have been reported in other qualitative work with expectant mothers [40].

Some of the women noted experiencing psychological stress. Energy-dense comfort foods may be used as a coping mechanism for general life stressors and anxieties, or an affordable alternative to other rewards which provides immediate gratification [41]. Examination of the interpersonal sphere of the SEM showed a strong influence among household members over dietary patterns. Similarly, parent relationships and number of children in the home were found to differently influence obesity risk related household practices among rural, low-income families [42]. Some mothers prioritized the diet of their children before their own, which can lead to undereating or altered consumption patterns. In turn, prolonged dysfunctional eating patterns may trigger psychological and physiological stressors [43].

Further investigation into the role low-income women have in grocery shopping and household food choice is needed to gauge whether their apparent level of control is accurate or overestimated. While women are perceived to be the gatekeepers of what a family eats, male partners often share responsibility and show interest in healthy food behaviors related to grocery shopping [44]. For some families, children were often present for grocery shopping and had considerable influence on what was purchased. In this study, and others, some mothers uphold certain “guidelines” for what is allowed in order to maintain healthy food options but allow for treats, snacks, and other exceptions that may undermine their intentions [45].

At the community level, concerns like lower accessibility to nutritious foods and restricted transportation options were not common obstacles brought up in the focus groups. This may be an example of a regional difference in barriers for low-income individuals. Rather, many participants stated they patronized a variety of area grocery stores and based their shopping location preferences on factors like sales, advertisements, and quality of specific food items instead of simply on convenience or proximity.

Health care professionals may have limited influence over food behavior. Several women in this study were hesitant to follow through on dietary advice from health care professionals like registered dietitians and physicians because they perceived the recommendations were not achievable. This breakdown in communication highlights the need for focusing greater attention on appropriate and effective education and messaging strategies [46]. Health care professionals may have more success promoting behavior change by soliciting information on past and current experiences with family and food to help individuals have greater success implementing dietary advice into their current life circumstance and cultural considerations [47,48].

Physicians receive minimal training in nutrition in general, let alone strategies to provide appropriate nutrition education for low-income populations. Survey research has shown that doctors often spend little time on nutrition counseling but are open to additional training so that they can be better equipped to talk to patients about the importance of diet [49].

Since women frequently sought nutrition information on the internet, there may be untapped potential in reaching audiences via this preferred format with online nutrition education programming or a hybrid of interactive online and in-person interventions [50]. This type of information delivery provides a novel approach that allows education organizers an outlet to cut through conflicting internet noise to deliver consistent messaging. In another Iowa sample of low-income White and Hispanic women, approximately 50% went to the internet first for nutrition information as well [31].

Utilization of food assistance programs was assessed as part of the policy level of the SEM. Other studies have reported that SNAP funds typically last only two to three weeks or less if there are children in the household [9]. In a similar low-income group of Arizona women, 34% reported running out of food before the end of the month most of the time [51]. This sentiment was shared by some Iowa focus group participants with frustration voiced regarding funds not lasting the entire month, wanting to purchase healthier foods but not being able to afford them, and restricted food offerings under SNAP and WIC guidelines. These criticisms fall in line with existing research on barriers to eating healthily with SNAP benefits, including the high cost of nutritious foods, inadequate program benefits based on income, and lack of nutritious options in stores [52]. Strategies proposed by Leung et al. to improve the diet of SNAP participants include incentives for nutrient-rich foods, restrictions on nutrient-poor foods, and biweekly funding instead of monthly to allow for better food budget management [52]. Not all eligible households participate in SNAP and other programs due to lack of knowledge about eligibility [53]. Additionally, participation in food assistance programs can be supported through environmental innovation to reduce stigma and support positive food relationships for the whole household [54].

Women are looking for ways to provide healthy, nutritious meals that their families will enjoy yet are economical, considering limited resources. This study, and others, show that of low-income mothers indicated that they often have knowledge of healthy diets but are challenged to implement those behaviors with their children [8,54]. Previous research by others indicates tailored nutrition education messages among low-income and low-literacy groups are most effective if focused on achievable positive behavior changes [41,46,48]. Messaging that includes behavioral and cultural constructs are the most beneficial for changing behaviors and directly influencing one’s life [52]. Motivators of healthy eating, such as looking and feeling better, setting a good example for their children, and feeling good about themselves may be more empowering messaging themes [55,56,57,58].

One of the strengths of this study is using qualitative methods with low-income women who may have limited time or misgivings about other research methods [55]. The focus groups allowed for flexibility in expressing views that would not have been possible with a survey alone and encouraged an open and personalized discussion. Participants shared intimate life circumstances, experiences, and personal challenges that they faced, such as chronic illness. These details shed light on the spectrum of barriers that prevent people from applying learned nutrition knowledge to daily practice. They also underscore the need to look at influences in disaggregated contexts and not only national trends to guide policy interventions and nutrition messaging. The long-term goal is to use this information, framed by the congruence between knowledge, practices, and perceptions, to guide realistic change in dietary patterns to support better health for low-income women and their families.

Limitations to this study include a small sample size for survey data from a narrow geographic region in Iowa. These results cannot be generalized to other low-socioeconomic populations. However, they do provide insights on the perceived influences on food choice for low socioeconomic women that can be expanded upon to develop interventions.

## 5. Conclusions

This research explored knowledge of healthy food choices in low-income women and their views about their ability to put their nutrition knowledge into practice. The objective was to evaluate the congruence between knowledge, perceptions, and practices to determine how to best to frame effective nutrition education and messaging based on real-world constraints. Many participants appeared to be aware of the relationship between health and diet and were confident in their ability to eat healthy food, but felt their circumstances limited potential action. The most common obstacles included time and inconvenience, family preferences and taste, and cost. There was a recurring perception that healthy food is more expensive and therefore less attainable.

Intervention messaging aiming to improve low-income women’s diet and thus decrease disease risk should focus less on nutrition education, as this knowledge is already high. Instead, messaging to low-income women may be most effective if cost, accessibility, time-saving preparation, and family needs are a focus. Interventions such as cooking classes should address how to use healthy foods in easily adaptable ways that are quick, budget friendly, and account for current household preferences, tastes, and practices. In relation to chronic disease risk, mediations should instill the idea that small habit changes can produce significant positive health changes.

## Figures and Tables

**Table 1:** Demographic and household characteristics of low-income Iowa women (*n* = 36).

	$\overset{–}{X}$ ± SD
**Age in years**	34.7 ± 8.8
**Number of children**	1.3 ± 1.5
**Total household size**	3.9 ± 2.0
	%
**Race**	
White	61.1
African American	38.9
**Marital Status**	
Single/Divorced	69.4
Married/Cohabitating	30.6
**Education**	
High school, GED, or less	41.7
Some college or Associates	36.1
Bachelor’s degree or higher	22.2
**Employment Status**	
Currently employed	50.0
Unemployed	11.1
Homemaker/Student	25.0
Disabled	13.9
**Food Security Status**	
High food security	30.6
Low food security	36.1
Very low food security	33.3
**Food Assistance Program Usage** ^†^	
SNAP	47.2
WIC	36.1
Child Nutrition	30.6
Food Distribution	8.3
No Food Programs	11.4

† Could select multiple options.

**Table 2:** Health characteristics and risk factors of low-socioeconomic Iowa women (*n* = 36).

Self-Reported Health Status	%
Poor–fair	38.9
Good	36.1
Very good–excellent	25.0
**BMI Category**	
Normal 18.0–24.9	22.9
Overweight 25.0–29.9	17.1
Class 1 obesity 30.0–34.9	25.7
Class 2 + obesity ≤ 35.0	34.3
**Smoking Status**	
Never smoked	33.3
Successfully quit	
Current smoker	27.8
**Alcohol Consumption Frequency**	
Less than 2 times per year	44.4
1–3 times per month	36.1
1–7 times per week	19.4
**Percent of Total Calories from Fat**	
Less than 30%	16.7
Average 30–35%	19.4
High 36–40%	27.8
Very high 40–50%	36.1
**Dietary Fiber Intake**	
Less than 20 g per day	83.3
20 g or more per day	16.7
**Fruit and Vegetable Servings per Day**	
5 or more per day	30.6
Less than 5 per day	69.4

**Table 3:** Reported self-efficacy of low-income Iowa women to maintain a healthy diet (*n* = 36).

I Can Manage to Stick to Healthful Foods Even If:	Very Uncertain	Rather Uncertain	Rather Certain	Very Certain
I need a long time to develop the necessary routines.	0	13.9	63.9	22.2
I have to try several times until it works.	0	13.9	61.1	25.0
I have to rethink my entire way of nutrition.	2.8	19.4	58.3	19.4
I do not receive a great deal of support from others when making my first attempts.	2.8	25.0	52.8	19.4
I have to make a detailed plan.	0	16.7	52.8	30.6
Self-efficacy summary score (mean ± SD)		15.1 ± 2.6 (possible range 5–20)		

**Table 4:** Consumption frequency of individual food items from the Food Frequency Dietary Screeners by low-income Iowa women (*n* = 36; * significant difference by race).

**Fat Screener Foods**	**1 or Less Time per Month**	**2–3 Times per Month**	**1–2 Times per Week**	**3+ Times per Week**
Hamburgers, ground beef, meat burritos, tacos	11.1	16.7	30.6	41.7
Beef or pork, steaks, roast, ribs, or in sandwiches	19.4	36.1	25.0	19.4
Fried chicken *	41.7	30.6	19.4	8.3
Hot dogs, or sausage	33.3	44.4	16.7	5.6
Cold cuts, lunch meats, ham	33.3	30.6	11.1	25.0
Bacon or breakfast sausage *	36.1	36.1	11.1	16.7
Salad dressings	30.6	25.0	16.7	27.8
Margarine, butter, or mayonnaise on foods	11.1	27.8	13.9	47.2
Margarine, butter, or oil in cooking	8.3	13.9	13.9	63.9
Eggs	22.2	16.7	25.0	36.1
Pizza	22.2	33.3	33.3	11.1
Cheese or cheese spreads	13.9	19.4	30.6	36.1
Whole milk	66.7	8.3	8.3	16.7
French fries, fried potatoes	22.2	38.9	19.4	19.4
Corn chips, potato chips, popcorn, crackers	25.0	30.6	19.4	25.0
Doughnuts, pastries, cake, cookies	33.3	47.2	5.6	13.9
Ice cream	55.6	36.1	5.6	2.8
**Fruit, Vegetable, and Fiber Foods**	**<1 Time per Week**	**1 Time per Week**	**2–3 Times per Week**	**4+ Times per Week**
Fruit juice, like orange, apple, grape *	30.6	8.3	25.0	36.1
Fruit fresh or canned	8.3	5.6	33.3	52.8
Vegetable juice, like tomato, or V-8,	61.1	8.3	13.9	16.7
Green salad (like lettuce or spinach)	22.2	16.7	38.9	22.2
Potatoes, incl. baked, mashed, fries	11.1	19.4	47.2	22.2
Vegetable soup or stew	30.6	41.7	11.1	16.7
Any other vegetables (peas, tomatoes, corn)	11.1	19.4	30.6	38.9
Fiber cereals like raisin bran, shredded wheat	63.9	13.9	16.7	5.6
Beans, e.g., baked, pinto, kidney, lentils	33.3	13.9	36.1	16.7
Dark bread such as whole wheat or rye	36.1	13.9	30.6	19.4

## Appendix A

**Table A1:** Logistic regression model of predictors of food security status among low-income Iowa women (*n* = 36).

			95% Confidence Interval for Odds Ratio		
	B (SE)	Sig.	Lower	Odds Ratio	Upper
Self-efficacy score	0.884 (0.393)	0.025	1.119	2.420	5.232
Household annual income		0.574			
<$10,000	−1.205 (3.104)	0.698	0.001	0.300	131.485
$10–24,999	−2.586 (3.268)	0.429	<0.001	0.075	45.581
$25–49,999	3.435 (4.084)	0.400	0.010	31.023	92,941.973
Race	6.422 (4.049)	0.113	0.220	614.993	1,719,462.786
Marital status	2.729 (2.406)	0.257	0.137	15.312	1711.076
Education level High School/GED		0.134			
Some college	−8.533 (5.726)	0.136	<0.001	<0.001	14.732
Bachelor’s+	−10.738 (5.761)	0.062	<0.001	<0.001	1.739
Employment	6.719	0.043	1.238	828.243	553,910.691
Cigarette smoking	4.757 (3.345)	0.155	0.165	116.348	81,828.744
Children in household	1.824 (2.061)	0.376	0.109	6.198	352.119
Constant	−18.102 (8.519)	0.034		<0.001	
Percent correct	Food secure		91.7	Overall	88.6
	Food insecure		81.8		
Model significance	*p* = 0.031				
